# Are There Age-Related Differences in the Ability to Learn Configural Responses?

**DOI:** 10.1371/journal.pone.0137260

**Published:** 2015-08-28

**Authors:** Rachel Clark, Michael Freedberg, Eliot Hazeltine, Michelle W. Voss

**Affiliations:** 1 Interdisciplinary Graduate Program in Neuroscience, University of Iowa, Iowa City, IA, United States of America; 2 Department of Psychological and Brain Sciences, The University of Iowa, Iowa City, IA, United States of America; 3 Aging Mind and Brain Initiative (AMBI), The University of Iowa, Iowa City, IA, United States of America; Center for BrainHealth, University of Texas at Dallas, UNITED STATES

## Abstract

Age is often associated with a decline in cognitive abilities that are important for maintaining functional independence, such as learning new skills. Many forms of motor learning appear to be relatively well preserved with age, while learning tasks that involve associative binding tend to be negatively affected. The current study aimed to determine whether age differences exist on a configural response learning task, which includes aspects of motor learning and associative binding. Young (M = 24 years) and older adults (M = 66.5 years) completed a modified version of a configural learning task. Given the requirement of associative binding in the configural relationships between responses, we predicted older adults would show significantly less learning than young adults. Older adults demonstrated lower performance (slower reaction time and lower accuracy). However, contrary to our prediction, older adults showed similar rates of learning as indexed by a configural learning score compared to young adults. These results suggest that the ability to acquire knowledge incidentally about configural response relationships is largely unaffected by cognitive aging. The configural response learning task provides insight into the task demands that constrain learning abilities in older adults.

## Introduction

The growing aging population presents a challenge because most people experience age-related decline in cognitive abilities that are important for maintaining functional independence, and successful aging requires the ability to learn new information to perform complex tasks. Intriguingly, some types of learning appear relatively preserved with normal aging, while others show dramatic decline, but the critical features that determine the extent to which aging affects learning are not yet known. Understanding these features is essential for both basic and applied research. From a basic science perspective, examining the task components that are primarily affected by aging will delineate the cognitive and neural systems that support different types of learning. From an applied perspective, characterizing the particular deficits that occur with aging will assist the development of better tools and therapies for older adults.

The goal of this study is to better specify age-related declines in learning processes by examining age differences in configural response learning. Configural responses require coordinated motor control between multiple effectors (such as fingers). Learning covariations between cues and configural responses involves an interaction of motor learning, associative processing and acquisition of statistical regularities. While many forms of learning involving motor skills and simple deterministic regularities are relatively preserved with aging [1, 2], there is strong evidence that associative binding and probabilistic learning processes decline with age [1, 3, 4].

In this way, configural learning lies at the nexus of two general themes in what is known about how aging affects learning. On the one hand, because configural response learning reflects the learning of a motor skill [5] and some aspects of motor skill learning are unaffected by age [2], one might expect configural response learning to be preserved. On the other hand, because configural response learning requires the binding of associations between specific stimulus-response pairs [5], one might predict that older adults show less learning than young adults. In support of this prediction, Stillman and colleagues [4] found age-related deficits in the ability to form probabilistic associations within sequences, even at the level of first-order regularities, which were previously thought to be impervious to aging. Given the combination of motor skill learning and associative binding, our configural learning task represents a unique and informative test case to extend this literature. Our prediction was that the requirement of associative binding would be sufficient to result in age-related differences in configural response learning, and therefore older adults would perform reliably worse than young adults on a configural response learning task.

### Sequence learning

Age differences in acquiring knowledge of statistical regularities through motor learning have been primarily studied with the serial reaction time task (SRTT) [6]. In this task, participants respond to stimuli on the screen with corresponding key presses. Unbeknownst to the participant, the stimuli repeat in a predetermined sequence. Therefore, although the participant may not be aware, there are contingencies between sequential events. Later in the task, the stimuli appear in a random order. Learning in the SRTT is operationalized as either increased accuracy or shorter reaction times (RTs) for predictable stimuli compared to random stimuli [7].

Young and older adults generally demonstrate a similar ability to learn deterministic sequences in the SRTT, such that each event or previous two events could perfectly predict the subsequent event [8–12]. This age-invariance is not surprising in some respects, given previous evidence that many aspects of simple deterministic motor learning are preserved in older adults [1, 2, 13, 14]. Moreover, it has also been shown that older adults can learn first and second-order deterministic perceptual sequences in tasks with minimal motor requirements [11]. However, when the sequence includes a probabilistic component such that a given event *probabilistically* predicts subsequent events, older adults tend to perform worse than young adults [1, 4]. One explanation for this pattern of results is the greater demand of associative binding in probabilistic sequence learning [4].

### Associative binding

Results from experimental paradigms generally support the proposal that older adults are less able than young adults to encode and utilize covariation to make associations [3, 15]. Researchers have typically examined associative binding by simultaneously presenting pairs of stimuli and then testing participants’ ability to remember the specific stimulus pairs compared to either individual items or recombinations (i.e., reconfigurations) of the stimuli that form reconFigd pairs [3, 16–20]. Results generally show that older adults are disproportionately worse at associative binding between features, items and their temporal context, and forming inter-item associations in both intentional and incidental encoding [16–18, 21]. These results are consistent with the associative deficit hypothesis (ADH) proposed by Naveh-Benjamin [3], which describes an age-related deficit in the associative binding of “mental codes” compared to relatively better performance on recall and recognition of single items. The deficit is also highlighted by the greater difficulty older adults have in correctly rejecting reconfigured pairs compared to studied pairs, and this may stem from a deficit in recollection of context-specific representations [18, 20]. This associative deficit has been proposed to apply under both explicit and implicit task instructions [16, 22], during probabilistic sequence learning [4], and has been generalized to nonverbal stimuli, such as binding faces with scenes [18] and binding face pairs [20]. Because configural learning requires the binding of the relationship *between* response elements (e.g., fingers) over and above stimulus-response associations for individual elements [5], we reasoned that the associative deficit should also apply to inter-response relationships acquired during configural response learning.

### Configural Response Learning

Configural response learning involves learning to simultaneously arrange multiple responses (such as finger presses) to form a single unified response. This requires not only the execution of individual movements, but also the configuration of movements in relation to each other [5, 23], involving both motor skill learning and associative binding. Configural learning is involved in many aspects of healthy aging such as adapting to a new car or learning new activities such as musical instruments, dance, or sport. However, little is known about whether there are age differences in this type of learning. Hazeltine and colleagues [5] compared the performance of frequently practiced configural responses to both reconfigured and novel configural responses and found that individuals formed associations between the elements of a particular configural response (i.e., a “chord” such as a piano chord) rather than simply strengthening the ability to map individual stimuli to responses. Learning also appeared to be implicit because the performance benefits were observed without subjects explicitly encoding which stimuli are likely to co-occur [5].

In addition, the task demands of the configural learning task and its relationship to the widely studied SRT task provides some leverage for predicting the nature of age-related learning deficits. Like the SRT task, the configural response task is thought to primarily assess implicit motor learning processes [5]. However, the configural response task does not require participants to hold information in time to form the associations necessary to improve performance. Also in contrast to the SRT task, binding must occur between simultaneously presented stimuli or simultaneously produced responses. Along these lines, the configural response task does not require participants to generate expectations about individual stimuli or key presses. All individual stimuli and key presses are equally probable on every trial so it is not possible to anticipate the production of an individual key press. Moreover, the perceptual and motor demands are greater than those in the SRT task, and previous work suggests that older adults demonstrate learning deficits with more complex tasks [24].

Thus, we predicted the requirement of simultaneous associative binding processing would result in older adults learning configural responses more slowly and less overall compared to young adults. In this case, older adults would respond more quickly on all trials as they practiced, but they would show less response combination-specific learning than young adults for stimulus combinations that were practiced compared with reconfigured stimulus combinations that were composed of equally familiar individual elements. This would indicate that older adults perform reliably worse than young adults in associative binding of multi-digit movement responses.

### Current Study

In our study, we modified the configural response task so that participants initially practiced stimulus-response mappings that involved matching a face stimulus with a single finger key press. After the mappings were encoded, faces were presented in pairs such that participants made two independent finger presses simultaneously to the two stimuli. Certain face pairs were practiced more than others and we measured the differences in response time and accuracy between frequently practiced and infrequently practiced pairs. The infrequently practiced pairs served as probes for the nature of associative learning because each face and finger press response considered individually was presented with equal frequency. Therefore, item familiarity remained equivalent for all items across frequently practiced and infrequently practiced pairs, which allowed us to specify configural learning as the difference in performance related to the encoding of the pairwise associations of simultaneously presented items unconfounded by familiarity with the individual stimulus-response associations. It follows that the faster response times for frequently practiced pairs relative to infrequently practiced reconfigured pairs, would be due to associative binding between the items and not familiarity of any specific item or response. Hazeltine and colleagues observed configural learning of this nature primarily in response times rather than accuracy [5], which also indicates the observed speeding of responses for frequent pairs is not due to speed-accuracy trade-offs in response times. Therefore, shorter response times to frequently practiced pairs relative to infrequently practiced pairs would indicate configural response learning. Thus, assessing the difference in performance between frequently practiced pairs and infrequently practiced reconfigured pairs enables us to test our hypothesis that older adults will perform worse than young adults in learning dependent on associative binding.

## Materials and Methods

### Participants

Twenty healthy young (M = 24.0 years, SD = 3.3; 9 F) and 20 healthy older (M = 66.5 years, SD = 4.8; 10 F) adults participated in the study in accordance with the University of Iowa’s Institutional Review Board’s (IRB) policies and procedures. All study policies and procedures were approved by the University of Iowa’s IRB. Participants were recruited from the greater Iowa City community using an approved University email advertisement, local fliers, and approved advertisements at the University of Iowa Hospitals and Clinics (UIHC). Eligible participants had to meet the following criteria: 1) demonstrate strong right handedness, scoring a 75% or above on the Edinburgh Handedness [25]; 2) be between the ages of 18 and 30 for young adults and between 60 and 80 years for elderly adults; 3) score greater than 24 on the MMSE-2SV [26, 27]; 4) have no self-reported psychiatric and/or neurological condition, including stroke or clinical depression; 5) have normal color vision; 6) have corrected visual acuity of 20/40 or above; 7) have no self-reported regular use of medication that could affect the central nervous system (e.g., psychotropics, recent or current chemotherapy, hypertension medication); and 8) sign a written informed consent. Young adults had an average of 16.5 years of education (SD = 1.8), whereas older adults had an average of 17.8 years of education (SD = 3.1), but this difference was not statistically significant (p = 0.11). Older adults also performed near ceiling on the MMSE-2SV with a mean score of 29.2 out of 30 (SD = .93). Musical experience did not differ between age groups (see *Age differences in musical experience* in supplemental materials, S1 Text).

### Configural learning task

#### Mapping phase

**Fig 1:**
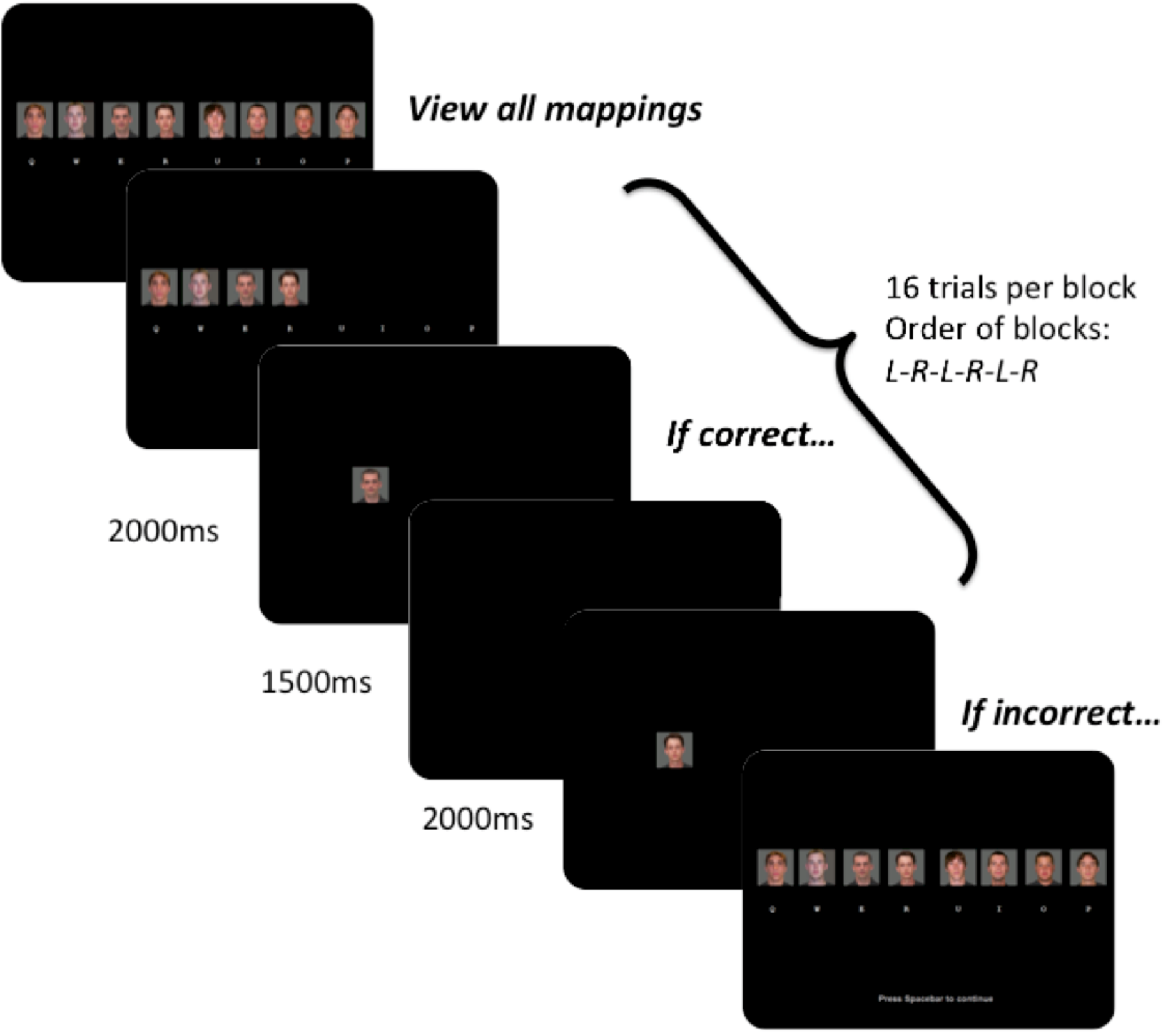
Initial stimulus response mappings phase: Example trial from the stimulus-response mappings phase that preceded learning trials. Letters under each face stimulus correspond to the letters on the keyboard mapped to each face stimulus (left hand: q, w, e, r; right hand: u, i, o, p).

Before starting the learning phase of the task, participants learned eight specific face-finger combinations (see Fig 1). During this mapping phase, participants responded to a single 100 x 100 pixel face presented on a computer screen. Four of the faces were mapped to the four non-thumb fingers of the left hand, and the remaining four were mapped to the four non-thumb fingers of the right hand. Participants responded on a standard ‘qwerty’ keyboard using the ‘q’, ‘w’, ‘e’, and ‘r’ buttons for the left hand, and the ‘u’, ‘i’, ‘o’, and ‘p’ buttons for the right hand. Each trial began with the presentation of a fixation cross for 500 ms, followed by an individual face on either the left or right side for 2000 ms, followed by a 1000 ms inter-trial interval (ITI) (see Fig 1). Participants were instructed to press the correct key response when the face appeared. Each time they pressed an incorrect response, feedback was given by presenting the full array of eight faces, which indicated the correct responses for all eight faces. Participants completed 6 blocks that alternated between left- and right-hand only response blocks (L-R-L-R-L-R; cued by a slide showing only the stimuli of the appropriate hand for the given block). Each block during this mapping phase contained 16 randomly ordered trials during which each face stimuli on the right or left was presented four times. The mapping phase lasted approximately 7 minutes. There were no age differences in the ability to learn the initial mappings (see *Mapping phase performance* in supplemental materials, S1 Text).

#### Learning Phase

**Fig 2:**
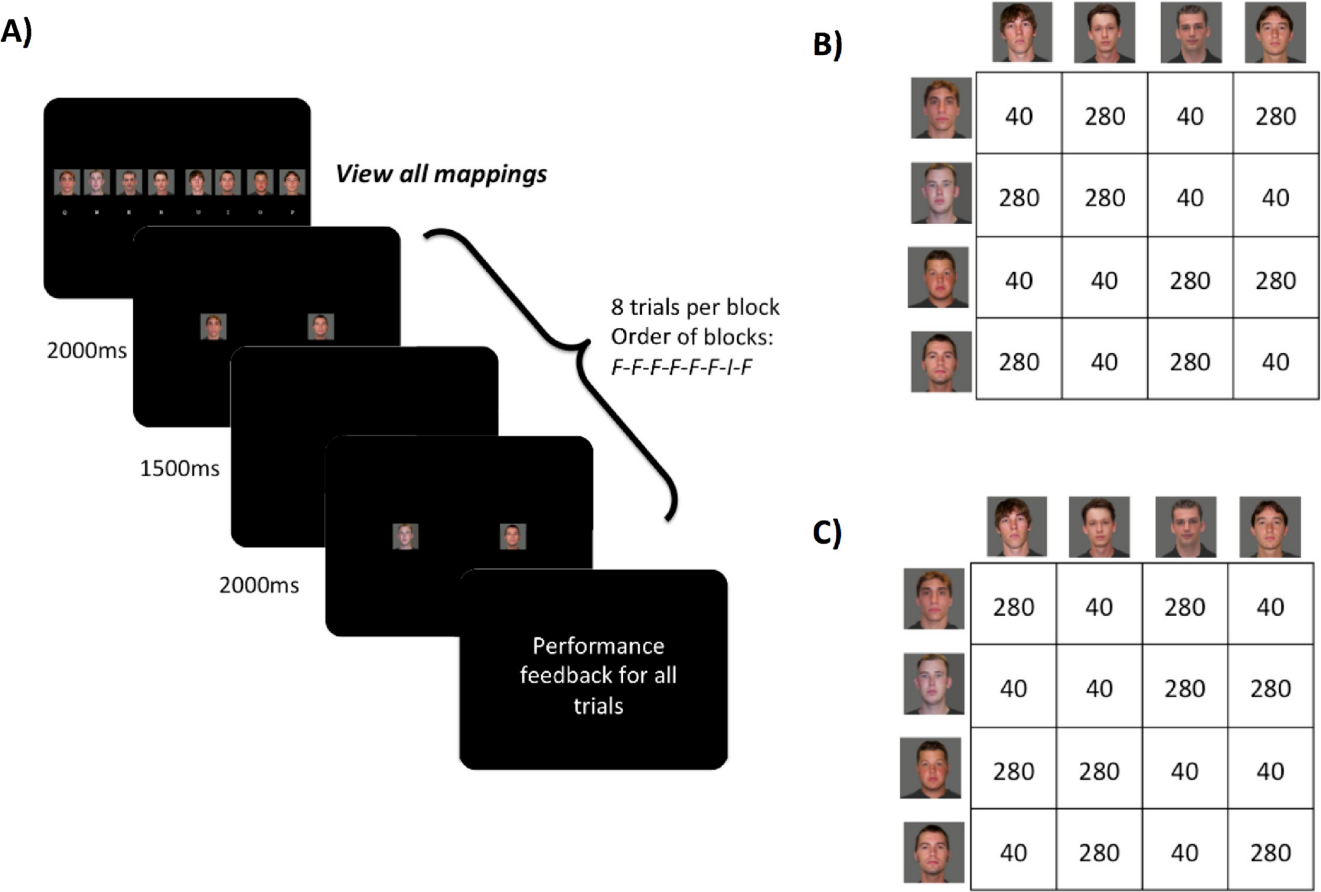
(A) Example trial sequence for one session of configural learning trials. Letters under each face stimulus on the first screen correspond to the letters on the keyboard mapped to each face stimulus (left hand: q, w, e, r; right hand: u, i, o, p); (B and C) Matrix of the two possible response mappings (i.e., for counterbalancing purposes), where the number in each cell refers to the frequency at which the specific face-face pair appeared in the task for a given subject (280 = Frequent trial pairing and 40 = Infrequent probe trial pairings). The numbers in the cells demonstrate that in both response mapping sets (B and C), a given face is seen 640 times (i.e., sum of any row or column) and thus all individual response elements are equally familiar across the learning task.

During the learning phase participants continued the task on the same computer used during the mapping phase. Participants again responded using the four non-thumb fingers of each hand on each trial. The same faces that were used during the practice phase were presented simultaneously to the left and right of a central fixation (see Fig 2). That is, on any given trial, one face from the left set and one face from the right set were presented. Participants responded with left and right finger responses (as simultaneous as possible, see *Response asynchronies* in supplementary materials (S1 Text) for more information) based on their learned mappings (see Fig 2). The 4 left-hand responses and 4 right-hand responses combined to create 16 total possible face-face pairs. Eight of those pairs were assigned to be frequently practiced (FR) pairs, and the other 8 were infrequently practiced (IF) (see Fig 2). As described above, this strategy was taken to ensure that each face stimulus appeared an equal number of times so that familiarity of individual response elements was matched across FR and IF blocks; thus the IF blocks served as probes to assess configural learning of specific face-face pairings. FR and IF face pairs were counterbalanced across participants.

Each block consisted of 8 trials; stimuli were sampled randomly without replacement from either the 8 FR pairs (blocks 1–6 and 8) or the 8 IF pairs (block 7). The blocking of frequent pairs and infrequent probe pairs is similar to blocking of random response elements in probe blocks for the SRT task [8,28], is similar to the blocked probe trials for the configural learning task in a previous study [5], and facilitates the direct transfer of the experimental design to an fMRI blocked design task in future studies. Trial timing was the same as mapping trials (500 ms fixation, 2000 ms trial duration, 1000 ms ITI). A 4000 ms fixation screen occurred after each set of 8 trials, signaling the end of a block. Seven blocks of FR pairs and 1 block of IF pairs made up one session. Each session lasted approximately 6 minutes, with a brief 1-minute break between sessions. After each session, feedback in the form of average performance (response time and accuracy) was presented to the participant (see Fig 2). During each of two visits to the lab, participants completed 5 sessions for a total of 280 frequent trials and 40 infrequent trials, which lasted a total of 40 minutes; the second visit was approximately 7 days after the first visit. For counterbalancing purposes, all participants were randomized to one of two response set mappings (see Fig 2B and Fig 2C). Face stimuli were chosen from young adult male neutral faces in the Center for Vital Longevity Face Database [29, 30].

### Data Analysis

Trials in which either the left or right response was incorrect were considered errors. For the remaining trials, we used the maximum RT between the left and right responses as the trial RT. For block-wise analysis of improved performance, we analyzed average trial RTs for correct frequent trials for each FR block across all sessions. To measure configural learning, trial RTs in blocks 1–6 and block 8 were averaged to obtain a RT measure for FR pairs for each session, and trial RTs in block 7 were averaged to obtain a RT measure for IF pairs for each session. Because there were only 8 trials of IF pairs per block and 1 IF block per session, subjects were excluded if they did not respond correctly to any IF trials for 2 consecutive blocks or for more than 2 blocks total across both days.

To assess configural learning, we computed a *configural learning score* that represents RT to FR pairs compared to RT for IF pairs. This configural learning measure captures the specific difference for the association between two responses rather than general improvement in individual stimulus-response mappings or the ability to combine any two responses. We calculated this measure for *each session* using the following equation:

$$\frac{\left(AvgRT_{IFpairs})-(AvgRT_{FRpairs}\right)}{Avg(SD_{RT_{IFpairs}},SD_{RT_{FRpairs}})}$$

In this way, higher scores reflect faster RTs for FR pairs relative to IF pairs, and each individual’s score is normalized by their average variability in RT [31]. Changes in this measure across sessions indicated combination-specific learning.

### Statistical Analysis

All statistical analyses were performed using R (version 3.1.0) and SPSS. To assess motor-skill performance improvement differences between groups (young vs. older), RTs for frequently performed pairs were submitted to a non-linear mixed effects model using R’s non-linear mixed-effects (NLME) package. Mixed effects modeling was selected over repeated-measures ANOVA as our statistical technique for the purpose of more accurately modeling individual differences. Because ANOVAs cannot separate random effects (e.g. inter-subject variability) from fixed effects (such as effects of age differences), mixed-effects modeling is a more powerful statistical analysis technique [32, 33]. To detect possible differences in configural learning across sessions for each day, configural learning scores were submitted to linear mixed-effect models using R’s “lmerTest” and “lme4” packages. Accuracy for each condition (FR, IF) across sessions was also analyzed with a linear mixed-effect model with same procedures as described for configural learning. The rationale for using two different statistical analyses was to better capture the specific pattern of results for each analysis (frequent RTs, configural learning scores, accuracy). For example, incidental learning of practiced responses over training at the resolution of blocks is well modeled using a 3-parameter asymptotic exponential [34]. Thus, because frequent pairs were practiced continuously over each session, we modeled motor-learning performance in this task using the same method. On the other hand, the observed configural learning scores in our experiment generally followed a linear path across sessions (and could not be measured at the resolution of blocks). Accuracy analyses also followed a linear trajectory across sessions and were modeled across sessions to directly compare with configural learning scores. Thus, linear modeling of these data (configural learning scores, accuracy) was more appropriate than using a 3-parameter asymptotic exponential. Because these procedures separate random effects from fixed effects, the model-predicted fits (including the ones illustrated in our figures) will not include random effects. For statistical testing, effects are considered statistically significant if *p*-value is less than 0.05.

#### Modeling accuracy

Accuracy for frequently and infrequently performed pairs was modeled using a linear-mixed effect model. Because these data are proportional, accuracy scores were transformed into logit space prior to analysis. This model was fit to session-specific performance using R’s [35] linear mixed-effects (lme4) package [36]. The starting model for each analysis included 1) fixed effects for *intercepts*, *linear slope*, and *quadratic slope;* 2) fixed effects of age group on *linear slope* and *quadratic slope* and 3) random subject-specific effects on *intercept*, *linear slope*, and *quadratic slope*. Model-comparison procedures based on the Bayesian Information Criterion [37] were used to trim this complex model. Fixed effects for age group on a specific parameter were eliminated if the more complex model did not have a significantly better fit than the simpler model. Random effects for specific parameters also were eliminated if the complex model did not have a better fit, which would indicate that parameter estimates did not vary significantly across participants and that only a fixed-effect estimate would be necessary. The time variable (session) was centered so that the linear slope for each group was calculated at the midpoint of each day (session 3).

#### Modeling motor-skill performance

RTs for frequently performed responses were modeled using a three-parameter exponential function using block as our time variable for each day. In the asymptotic exponential function, *a* indexes the asymptote for learning, *b* indexes the change in RT from the initial block to the asymptote (i.e., magnitude of overall RT change), and *c* indexes the rate of change.

This model was fit to block-specific performance using R’s non-linear mixed-effects (NLME) package [38]. The starting model for each analysis included 1) fixed-effect intercepts for *a*, *b*, and *c;* 2) fixed effects of age group on *a*, *b*, and *c*; and 3) random subject-specific effects for *a*, *b*, and *c*. Model-comparison procedures based on the Bayesian Information Criterion [37] were used to trim this complex model. Fixed effects for age group on a specific parameter were eliminated if the complex model did not have a better fit than the simpler model. Random subject-specific effects for specific parameters also were eliminated if the complex model did not have a better fit, which would indicate that parameter estimates did not vary significantly across participants and that only a fixed-effect estimate would be necessary. Starting values for the nlme procedure were determined by fitting a 3-parameter exponential function to the data using the nonlinear least-square procedure (nls) [39].

#### Modeling configural learning scores

Because infrequent pairs were acquired only once per session (for a total of five blocks per day), we calculated the learning scores for each session using Eq 1 and then fit a linear mixed-effect model to these learning scores. The modeling procedure for learning scores mirrored the analysis for accuracy (see above). The linear model included fixed effects for *intercept*, *linear slope* (the rate of change over session) and a *quadratic slope* (the rate of acceleration or deceleration of learning score across sessions). The *quadratic slope* was calculated for each day to determine whether a significant acceleration or deceleration in learning score occurred across all 5 sessions. The *linear slope* determined the rate of change in learning score across sessions for each day and was centered at the midpoint of the five sessions (session 3). Random effects of subject on *intercept*, *linear slope*, or *quadratic slope* were discarded if likelihood ratio tests revealed they did not contribute significantly to the modeled variability similar to the accuracy analysis.

## Results

Two subjects from the older age group were excluded from the analyses on the basis of three or more missing blocks of data (N for older = 18; M = 66.3 years, SD = 4.1; 10 F; 17.9 years of education, SD = 3.1). Two additional subjects were missing data from two or fewer non-consecutive blocks due to technical failure or wrong finger placement on the keyboard. Model-predicted RT and accuracy for the missing blocks were interpolated from surrounding blocks.

### Accuracy analysis

**Fig 3:**
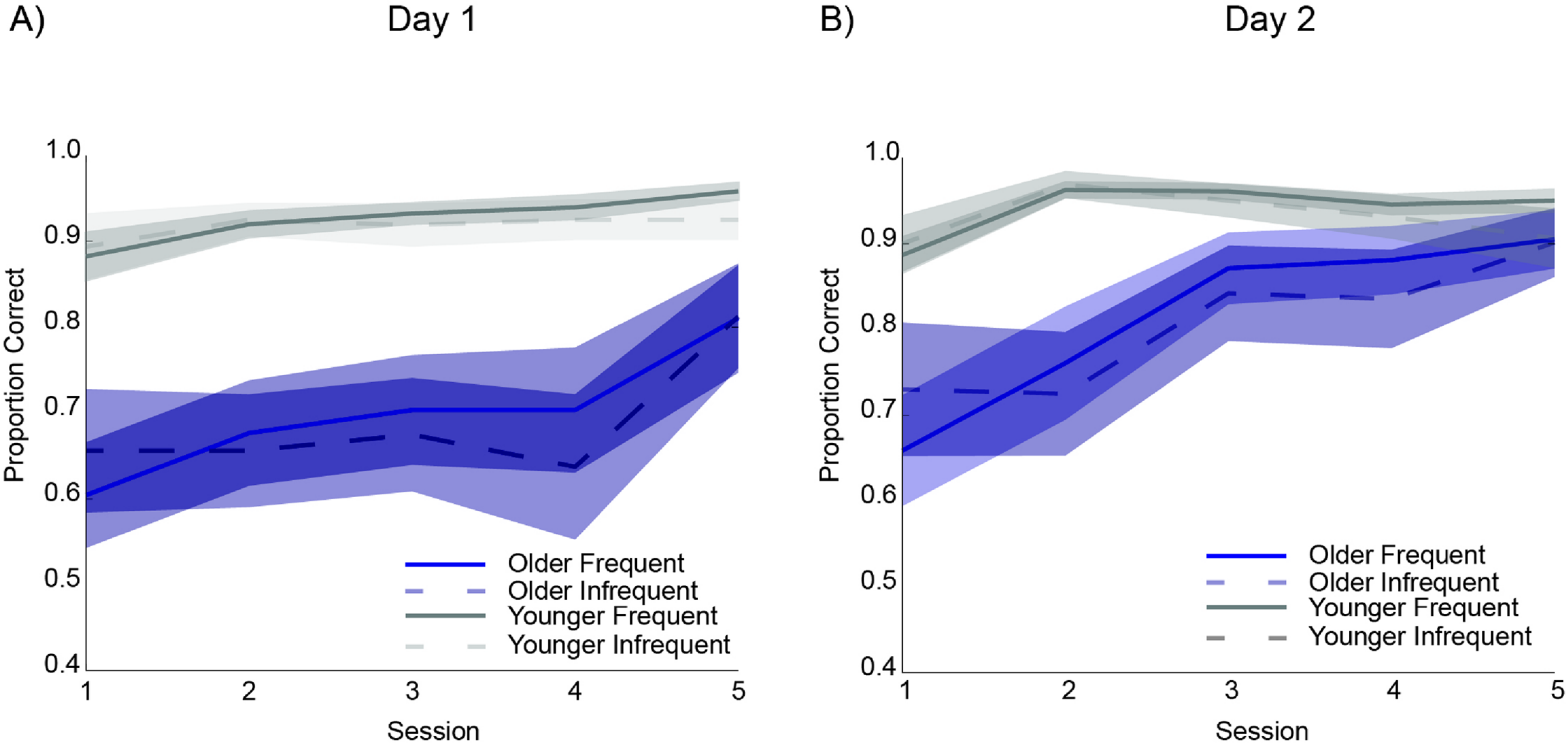
Proportion correct for frequently performed (solid lines) and infrequently performed (dotted lines) pairs across session for (A) day 1 and (B) day 2. Shaded regions represent standard error. Note in linear model, the time variable (session) was centered so that the linear slope for each group was calculated at the midpoint of each day (session 3).

Accuracies for each condition (FR, IF) across each session and day were submitted to a linear mixed-effect model after logistic transformation. The maximum random effects model justified by the data included a random effect for intercept, linear slope, and quadratic slope for participants. Significant main effects were detected for day (t(690.8) = 6.909, p < 0.001), linear slope (t(456.9) = 2.368, p < 0.05), age group (t(880.0) = 4.807, p < 0.001), but not pair frequency (t<1). These results indicate that 1) accuracies were higher on day 2 than day 1, 2) participants improved their response accuracy for both conditions within each day, 3) young adults achieved higher accuracies than older adults in general, and 4) accuracies did not differ between frequent pairs (FR) and infrequent pairs (IF) (see Fig 3). In addition, a significant quadratic effect was detected (t(690.7) = 2.419, p < 0.05), indicating that the overall rate of increase in accuracies decreased as each day progressed.

The accuracy analysis revealed two significant interactions. First, we observed a significant interaction between day and age group (t(690.8) = -3.108, p < 0.01) indicating that overall older adults had higher accuracies on day 2 than day 1, and this difference was significantly greater than the change across days for young adults. Second, there was also a significant interaction between day and quadratic slope (t(691.3) = -2.809, p < 0.01), indicating that the rise in accuracy across sessions diminished significantly more rapidly on day 2 than on day 1. No other interactions were significant. Because the interaction between age group and pair frequency was not statistically significant, this result provides a foundation for assessing *configural learning* as a relative speeding of RT in frequently practiced pairs compared to infrequently practiced pairs, unconfounded by accuracy differences between the two conditions.

In sum, young adults performed more accurately relative to older adults through day 2. Moreover, young adults were quicker to reach asymptotic performance, which occurred early on day 2. In contrast, older adults continued to show increases in accuracy through day 2. However, for both age groups, accuracies were not statistically different between FR and IF conditions across sessions. That the configural learning effect is not apparent in accuracy scores is consistent with Hazeltine’s previous study [5], and this supports the assessment of *configural learning* as an RT difference between FR and IF conditions unconfounded by differences in accuracy between the FR and IF conditions.

### Motor-skill performance (analysis of frequently performed pairs)

**Fig 4:**
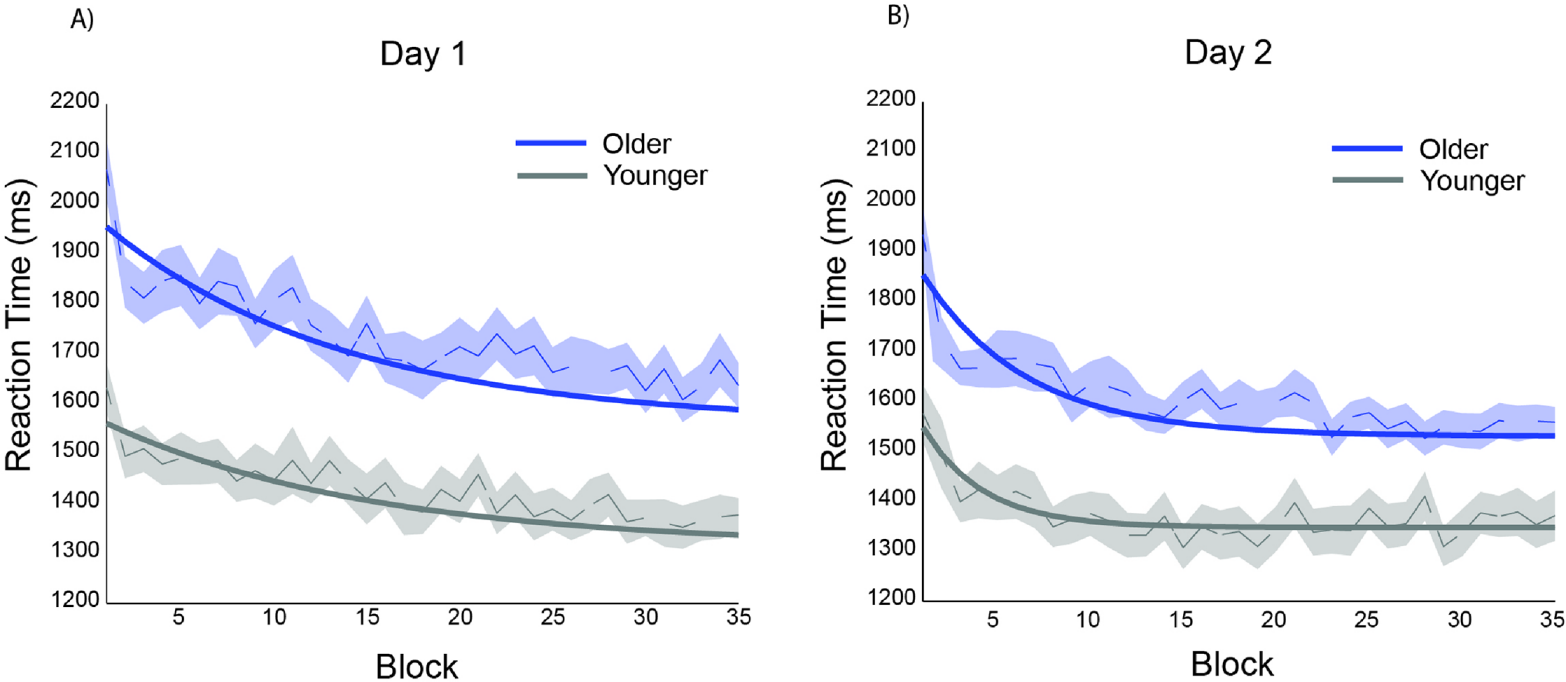
Reaction times for frequently performed pairs on (A) day 1 and (B) day 2. Only frequently performed pairs are modeled, rather than the comparison of frequent and infrequent pairs; this served as a method for assessing motor skill learning (basic response speeding) separately from configural learning. Participants performed 7 blocks of frequent pair responses in each of 5 sessions per day, which are plotted sequentially in the figure above as blocks 1 through 35 for Day 1 and Day 2. Dotted lines represent block-wise group averages; shaded region represents standard error; solid lines represent model-predicted values.

The maximum random effects model justified by the data included random effects for asymptote, the magnitude of RT decrements, and rate of RT change for participants. On day 1, significant differences were observed between groups for the asymptote (t(1310) = - 4.85, p < 0.001), and magnitude (t(1310) = -2.62, p < 0.01), but not rate of change (t(1310) = -0.61, ns). As illustrated in Fig 4, these results indicate that both groups showed statistically significant reductions in RTs across blocks on day 1. The young adults were approaching an asymptote that was 284 ms shorter than the older adults, but the older adults showed significantly greater reductions. Despite these differences the groups were similar in their speed to reach asymptote on day 1.

Since the 3-parameter model is best estimated per day rather than across days, a separate model was estimated for day 2. Significant performance gains were also observed on day 2; the asymptote (t(1304) = 58.95, p < 0.001), magnitude of RT decrements (t(1304) = 8.66, p < 0.001), and rate of change (t(1304) = 7.75, p < 0.001) across groups differed significantly from 0. Differences between groups were observed for asymptote achieved (t(1304) = - 3.54, p < 0.001), magnitude (t(1304) = -2.03, p < 0.05), and rate of change (t(1304) = 2.15, p < 0.05). Similar to day 1, young participants approached a lower model-predicted asymptote than the older participants, but the older participants showed greater decrements in RT. However, unlike day 1, there was a significantly faster rate of RT change for older adults over young adults on day 2. One possible explanation for this pattern of results is that young adults may have continued to experience performance gains on day 2, whereas older adults may have quickly reached their asymptotic performance early in day 2. Alternatively, older adults may have experienced reduced retention on block 1 of day 2 which resulted in a steeper decrease in RTs over subsequent blocks (see *Retention and Savings* in supplementary materials (S1 Text)).

### Configural learning scores

**Fig 5:**
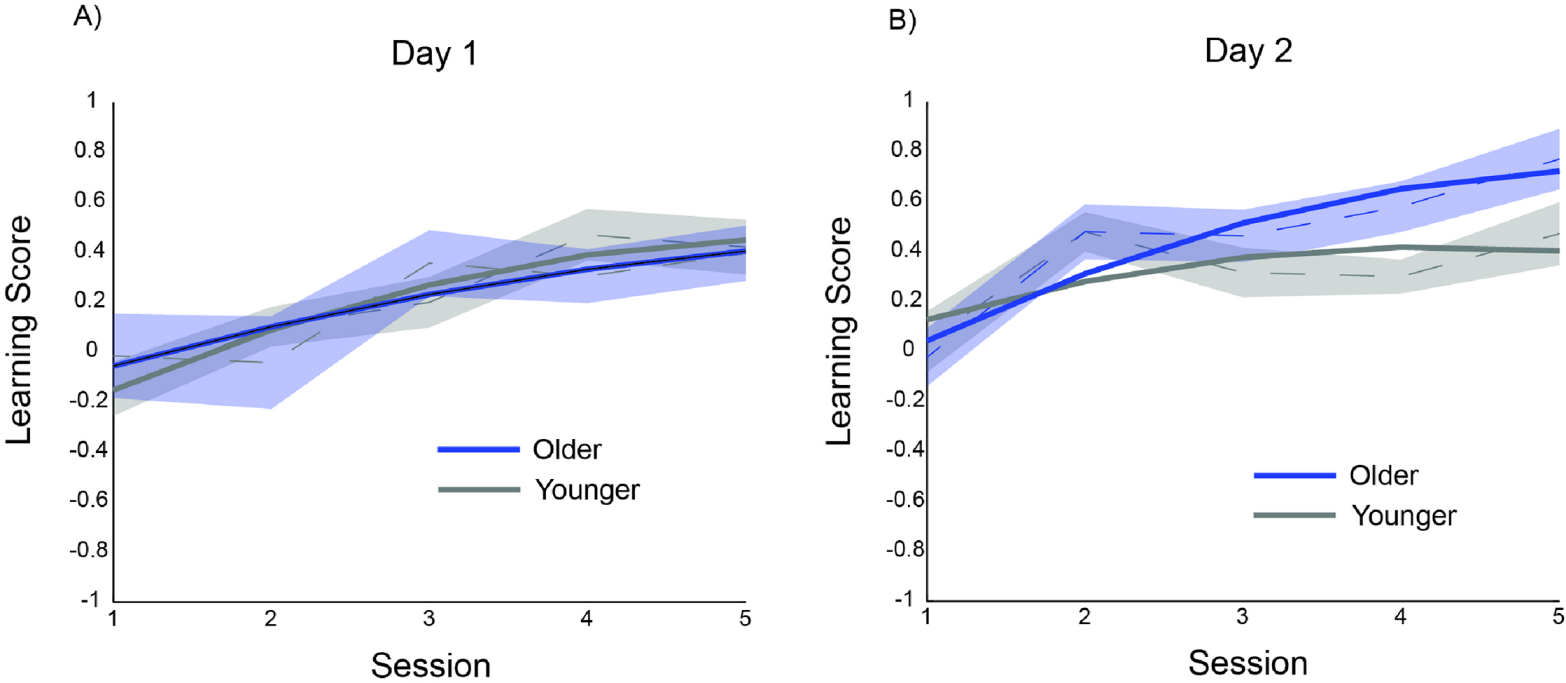
Observed and modeled configural learning scores plotted across session for (A) day 1 and (B) day 2. Dashed lines connect group average learning scores for each session; shaded regions represent standard error; solid lines represent model-predicted values. Note in linear model, the time variable (session) was centered so that the linear slope for each group was calculated at the midpoint of each day (session 3).

Configural learning scores were submitted to a linear mixed-effects model where the best-fit model representing the data included a random intercept for participant. Note that configural learning scores were computed such that a higher score indicated relatively faster response times for the frequently practiced pairs compared to infrequently practiced pairs. Learning scores were higher on day 2 than day 1 (t(332.8) = 2.691, p < 0.01). Significant learning was detected across sessions (t(333.50) = 2.804, p < 0.01). No quadratic effect or interaction between quadratic slope and day, or quadratic slope and group (ts < 1) was detected. Overall, these results indicate that configural learning followed a linear time course. This was also supported by a graphical comparison of the observed and modeled configural learning scores (see Fig 5). The only significant interaction was a three-way interaction between day, linear slope, and age group (t(333.7) = 2.056, p < 0.05), indicating that on day 2 older adults experienced a significantly larger rate of configural learning gains than young adults. One explanation for this effect is that young adults may have reached their asymptote for frequent pairs at the end of day 1, while older adults continued to improve, which contributed to a higher configural learning score.

In sum, although young adults performed more accurately on the configural response task through day 2 compared to older adults, accuracy did not differ between FR and IF conditions for either young or older adults across sessions (Fig 3). This provided a basis for measuring *configural learning* as a relative speeding of RT in frequently practiced pairs compared to infrequently practiced pairs. Not surprisingly, older adults were generally slower than young adults (Fig 4), however, the configural learning scores of older adults were similar to those of young adults (Fig 5).

## Discussion

The current study demonstrates that older adults can incidentally learn configural response relationships as well as young adults, indicating that the learning of some forms of associative encoding are relatively preserved. Although we observed age-related performance differences in the overall decrease in RT across the task, young and older subjects demonstrated equivalent configural learning rates; in fact, there was evidence that older adults were able to continue to reduce their RTs after the young adults reached asymptote. By examining learning across separate days, we characterized the extent to which age effects change as each age group approaches asymptotic levels of performance [40]. We conclude that while aging does affect the fluency of motor response (e.g., Fig 4), it does not negatively affect configural response learning (e.g., Fig 5). This suggests a dissociation of the ability to perform well on a task (fluency) and the ability to learn information based on experience (configural response learning).

Overall, our results are not consistent with our prediction that older adults would perform worse on the configural learning task because of a demand for associative binding among covarying events; there was no evidence that the older adults showed less ability to bind the distinct response components together than the young adults (e.g., Fig 5). Thus, the associative processing involved in forming relationships between motor responses may be separate from the cognitive mechanism involved in implicit learning tasks that may involve associative binding of other types of information [3,4]. Given the variability observed through studies of age effects on associative processing, and even different types of implicit learning [1, 41], it appears that separate types of processing, even with a commonality of implicit learning, rely on distinct brain regions or systems that are differentially affected by aging [42]. Interestingly, Reber and colleagues have recently introduced another variant of the SRT task called the serial interception sequence learning (SISL) task, which shows relatively large learning effects and very little evidence of explicit knowledge of learned sequences [43]. The task also shares some features of the configural learning task, such as learning across bimanual responses and temporal precision of responses. While Reber and colleagues have shown that patients with Parkinson’s Disease performed worse than healthy older adults and patients with mild cognitive impairment [44], it will be informative to examine whether there are effects of healthy aging on the SISL task in a sample of participants who also perform the configural response task presented here.

One potential limitation of our study is that we did not formally assess participants’ explicit knowledge of acquired configural responses in the task. For instance, some studies of sequential learning have tested participants on whether they could predict the next item in a sequence following learning [45–48]. This procedure assesses the nature of whether learned information is accessible through explicit retrieval or only manifests implicitly through changes in performance. We addressed this issue in a follow-up study (see *Role of explicit awareness* in supplementary materials (S1 Text)). Briefly, results showed that while most participants were aware of the frequency manipulation, their awareness was not correlated with learning. Although the sample for our follow-up study included only young adults, previous research supports that older adults tend to rely on familiarity during associative processing more so than young adults and it is very unlikely that explicit knowledge would be driving associative learning for older adults and not young adults [18–20]. Furthermore, learning in the configural response task cannot be enhanced by anticipating individual stimuli because all stimuli have an equal likelihood of appearance, therefore it is difficult to imagine how explicit knowledge could generate a configural learning effect in RTs and not accuracy as shown here. In addition, although some have argued that explicit awareness of acquired knowledge renders it difficult to operationalize learning as implicit [49], explicit knowledge of information learned under incidental encoding instructions may still be influenced by implicit memory representations [48, 49]. Overall, our results support the conclusion that individual differences in configural learning were not related to explicit awareness of the knowledge learned and can best be conceptualized as implicit learning. However, in future studies it will be fruitful to combine objective and subjective measures of conscious and unconscious representations acquired that could influence configural learning scores [48].

In the context of associative processing, it is interesting to note that based on the literature linking the hippocampus to associative binding [46, 50], we would expect the hippocampus to play a significant functional role during configural response learning. Indeed, across both short and long time spans, the hippocampus is required for forming associations between co-occurring items [51]. Given that the hippocampus is known to be negatively affected by aging [52], our results may lead to the prediction that configural response learning is a type of associative learning independent of hippocampal involvement as a way to explain the absence of age effects. However, other motor skill and implicit learning tasks have been shown to involve the basal ganglia [53–55]. Even though the basal ganglia also experience negative structural and functional changes with age [56, 57], older adults still tend to demonstrate intact motor skill learning on some varieties of these tasks [1, 11, 13]. Together, this begs the question: how do older adults learn configural response information as well as young adults? Results likely suggest that the cortical systems interacting with the hippocampus and the basal ganglia are a critical feature determining when and how normal aging affects learning. Functional neuroimaging provides a way to address this question, and more generally, provides a way to test theories linking learning systems in the brain to cognitive mechanisms of learning.

Given that effects of age most likely contribute to performance differences in RT (i.e., Fig 4), it is notable that the expression of configural learning did not differ between the age groups (i.e., Fig 5). Despite general slowing, older adults retained the ability to respond *selectively* faster on more frequently practiced pairs irrespective of overall repetition of individual response elements (i.e., sensitivity to response associations). This may reflect an independence between cognitive processes involved in performance versus learning. Future work may investigate the relationship between individual differences in components of configural response learning and performance on a validated neuropsychological battery sensitive to cognitive aging [58]. However, given the robust age effects on nearly all fluid cognitive abilities, our results suggest that the sensitivity to the effects of age for the cognitive processes involved in processing the to-be-learned information may not predict age-related decrements in learning per se.

## Supporting Information

S1 Text Supplementary methods and analyses. (DOCX)
