# Supplementary material for: Are There Age-Related Differences in the Ability to Learn Configural Responses?
Source: PLoS One. 2015 Aug 28;10(8):e0137260. doi: 10.1371/journal.pone.0137260 (PMC4552811; doi:10.1371/journal.pone.0137260)
Supplement: S1 Text — (DOCX) [file pone.0137260.s001.docx]

**Supplemental**

***Age differences in musical experience***

Given that the responses in our task resemble piano chords, we obtained measurements of musical experience to account for the effect of previous experience with configural postures. Of the 20 young adult participants, 14 individuals had played at least 1 instrument at some point in their life (70%) and eight participants had experience playing piano (40%). Of the 18 older adults (following exclusion), 13 individuals had played at least 1 instrument at some point in their life (72%) and nine participants had experience playing piano (50%). Aside from the number of years since participants had last played the instrument (young adults: mean 8.1 years; older adults: mean 45.2 years), musical experience did not differ between the two age groups.

***Mapping phase performance***

An unpaired t-test between younger and older adults revealed that there was a significant difference between groups in terms of reaction time on the unimanual blocks (t(38) = -5.179, p < 0.001), such that younger adults performed unimanual practice trials significantly faster than older adults. This result remained robust when the two excluded adults were discarded from the analysis (t(36) = 5.71, p <0.001). This result is unsurprising as young adults typically have faster RTs on cognitive tasks than older adults. We also identified a marginally significant difference in accuracy between younger and older adults (t(38) = 1.781, p = 0.083). This demonstrates a trend toward higher accuracies overall for younger adults (0.94) compared to older adults (0.85), though both groups performed with high accuracy. However, this difference did not persevere when the two excluded older adults were eliminated from the analysis (t(36) = 1.38, p = 0.18). These results indicate that both groups learned to successfully perform the unimanual face-finger mappings for the configural response task.

***Retention and Savings***

Retention and savings were calculated for each group using learning scores as well as accuracy for frequent chords only. The rationale for only analyzing accuracy of frequent chords is that the infrequent chords were performed on only one block per session. We examined retention by comparing task performance at the end of day 1 with the start of day 2, and savings by comparing the time required on day 2 to reach the final performance from day 1. This procedure allowed us to extend the total testing duration while minimizing any effects cognitive fatigue might have on the later stages of learning. Note that one additional participant was excluded from retention and savings analyses of learning scores due to incorrect responses on all infrequent pairs during session 1 of day 2.

Retention measures were calculated for each participant whereby performance for the last session of day 1 was subtracted from the first session of day 2:

$$\left( FR Accuracy Day 2 session 1 \right)-\left( FR Accuracy Day 1 session 5 \right)$$

$$\left( Learning score Day 2 session 1 \right)-(Learning score Day 1 session 5)$$

Retention measures of accuracy and learning scores were contrasted between age groups using unpaired t-tests. A significant difference between groups was found for retention in terms of accuracy (t(36) = 2.49, p< 0.05). This indicates that young adults retained relatively higher accuracy scores on day 2 compared to older adults. However, the unpaired t-test revealed no significant difference between groups in terms of retention of learning scores (t(35) = -0.14, p = 0.88). On average older adults dropped 0.38 learning score points from the end of day 1 to the beginning of day 2. Similarly, younger adults on average dropped 0.35 learning score points.

Savings measures were calculated for each participant as the number of sessions on day 2 needed to reach the learning score or accuracy the participant had achieved on the last session of day 1. Thus, we are defining savings as the time (in sessions) saved as a result of practice on day 1. No difference was observed between groups for savings of accuracy on frequent chords (t(36) = 0.25, p = 0.80). Similarly, no difference was detected (t(35) = 0.65, p = 0.52) between groups for savings of learning scores. On average older adults saved 3.24 sessions, whereas younger adults saved 2.85 sessions.

While older participants demonstrated less retention of configural responses (older adults’ accuracy scores decreased by more than young adults’ scores from the end of day 1 to the first session of day 2), both age groups retained similar levels of expressed learning (quantified by the learning score) between day 1 and day 2. We measured savings as a way to address whether young and older adults were able to use previously acquired associations to more quickly improve on the task at the second visit. There were no differences between the groups in terms of how quickly they returned to the achieved accuracy or learning score from the day before. Both young and older adults reached the end learning score from day 1 in approximately half the time on day 2. Thus, our results demonstrate an effect of age on retention of motor skill performance, but not on configural response learning. Measurements of retention and savings are often absent from studies of motor skill learning [for exceptions, see 1,2,3], particularly across multiple days.

***Age differences in response asynchrony***

Finally, we tested whether older and young adults differed in the timing of their bimanual responses across session and pair type. Response asynchronies (calculated as the difference in response times between the two responses on each trial) were submitted to a repeated-measures ANOVA using group (young vs. old), pair type (frequent vs. infrequent), session (1-5), and day as factors. The analysis revealed a significant main effect of session (F(4, 136) = 7.597, p < 0.001, ηp^2^ = 0.183), and session by group interaction (F(4, 136) = 4.803, p < 0.005, ηp^2^ = 0.124). No other main effects or interactions were significant. These results suggest that both groups were successful in decreasing their response asynchronies over the course of each day, but that older adults started with larger asynchronies on average compared to young. The absence of a main effect of age indicates older adults were not consistently more asynchronous than young adults across all sessions.

***Role of explicit awareness***

To provide information about the level of explicit awareness engendered during the task we conducted a follow-up study with young adults where following configural response learning participants were asked whether some pairs of faces were more likely to occur together than others. Of 16 participants, only 3 answered, “I really couldn’t tell” or “maybe.” All other participants responded, “yes, some faces were more likely to occur together.” Participants were then shown the 16 possible face pairs and asked whether the pair had appeared frequently or infrequently. On average, participants were 67% correct in identifying whether a certain face pair appeared frequently or infrequently. These results suggest that at least some of the information learned throughout the task is explicitly accessible. However, we did not find a significant relationship between accuracy on the follow-up questions and initial day 2 learning score (*r*=-.499, *p*=.071), final day 2 learning score (*r=*-.379*, p=*.140) or average of all day 2 blocks (*r*=-.400, *p*=.111), which is consistent with the idea that implicit learning and explicit retrieval mechanisms are independent [4].

**Supplementary References**

1. Rodrigue K, Kennedy K, Raz N (2005) Aging and longitudinal change in perceptual-motor skill acquisition in healthy adults. J Gerontol B Psychol Sci Soc Sci 60: P174-181.

2. Shea CH, Park JH, Braden HW (2006) Age-related effects in sequential motor learning. Physical Therapy 86: 478-488.

3. Voelcker-Rehage C (2008) Motor-skill learning in older adults—a review of studies on age-related differences. European Review of Aging and Physical Activity 5: 5-16.

4. Frensch PA (1998) One concept, multiple meanings: On how to define the concept of implicit learning: Sage Publications, Inc.
